# Pharmaceutical Applications of Glucose Syrup from High Quality Cassava Flour in Oral Liquid Formulations

**DOI:** 10.1155/2022/6869122

**Published:** 2022-01-24

**Authors:** Ivan K. Simpson, Frederick W. A. Owusu, Mariam E. Boakye-Gyasi, Philomena Entsie, Marcel T. Bayor, Kwabena Ofori-Kwakye

**Affiliations:** ^1^Department of Pharmaceutics, Faculty of Pharmacy and Pharmaceutical Sciences, Kwame Nkrumah University of Science and Technology, Kumasi, Ghana; ^2^Department of Herbal Medicine, Faculty of Pharmacy and Pharmaceutical Sciences, Kwame Nkrumah University of Science and Technology, Kumasi, Ghana

## Abstract

Pharmaceutical oral solutions are preparations in which the active ingredients are dissolved in suitable liquid vehicles such as syrups. This study sought to determine the potential of glucose syrup produced from high quality cassava flour (HQCF) as a vehicle or sweetener in the preparation of paracetamol syrup and simple linctus. Four formulations (two paracetamol syrups (B1 and B2) and two simple linctus formulations (A1 and A2)) were prepared using glucose syrup from HQCF as vehicle or sweetener while two controls (B3 and A3) were prepared for each group using sucrose syrup as vehicle or sweetener. Two brands of paracetamol syrup and simple linctus were purchased from retail pharmacies to serve as standards. Physical and organoleptic parameters such as pH, taste and color, microbial load, and drug content of all formulations were determined. All formulations passed the microbial load and drug content tests as specified by the British Pharmacopoeia. The paracetamol syrups were all sweet with characteristic bitter aftertastes except formulation B2 which was sweetened with sucralose. All the simple linctus formulations were sweet except A2 (sweetened with sucralose) which was very sweet. The taste masking capacity of the glucose syrup produced from HQCF matched that of the sucrose syrup in the products formulated. Therefore, glucose syrup from HQCF could be a suitable alternative to sucrose syrup as a vehicle or sweetener in oral liquid formulations and can ultimately reduce the cost of these oral liquid formulations.

## 1. Introduction

Glucose syrup is an undiluted hydrous liquid mixture of glucose, maltose, and other nutritive saccharides usually derived from edible starch by hydrolysis [1, 2]. Sugar syrups such as glucose and fructose syrups are utilized in the production and manufacture of fruit juices, brewery products, bakery products, confectionery products, pharmaceutical products, and candied fruits [2, 3]. The commonest raw material for producing glucose syrup is corn starch; however, due to the high demand for glucose syrup, nonconventional plants with potentially high yield for starch production such as wheat, millet, and sorghum have also been investigated in producing glucose syrup [3–5].

Cassava starch has also gained popularity as a potential starting material for glucose syrup manufacturing in the last decade, and it has been widely researched utilizing enzymatic, acid hydrolysis, or catalysts such as poly(4-vinylpyridine) hydrochloride (PVP, HCl), green clay activated with HCl, Amberlyst, Montmorillonite KSF, and zeolite [6–9]. Apart from starch, the industrialization of cassava has not seen any diversification until the introduction of high quality cassava flour (HQCF). High quality cassava flour (HQCF) can be defined as the ultrafine flour obtained from wholesome and newly harvested cassava roots that have been rapidly processed or the fine unfermented flour produced from the quick processing of freshly harvested cassava roots or simply as unfermented cassava flour, usually whitish or creamy in color, odourless, bland or sweet, and free from adulterants, insect infestation, sand, peel fragments, dust, and other impurities [10–13]. High quality cassava flour (HQCF) is a viable alternative to cassava starch in several industrial applications and can serve as the backbone of a cassava-based industry. In comparison to cassava starch, HQCF processing is less capital intensive and needs less inputs to be effective. In a variety of industries, including adhesives, sugar syrups, and synthetic alcohol, HQCF may be used as a starch substitute [1, 11, 12]. However, extensive research has not been carried out on the potential of HQCF as a starting material for glucose syrup and utilization of the syrup produced as a vehicle in liquid oral pharmaceutical dosage forms. In Pediatrics, one means of ensuring compliance with pharmaceutical liquid oral preparations is the inclusion of syrups as vehicles and sweeteners with preference for syrups derived from natural sources [14].

In Ghana, cassava (*Manihot esculenta*) is locally grown and available all year round. However, there is little use of its products in the local pharmaceutical industry especially in the production of oral liquid dosage forms. This research seeks to determine the suitability of glucose syrup obtained from HQCF as a vehicle or sweetener in the production of paracetamol syrup and simple linctus.

## 2. Materials and Methods

### 2.1. Materials

The materials are as follows: SEBStar HTL Alpha Amylase (Enzyme Innovation, California, USA), Diazyme X4 (DuPont/Danisco, lllinois, USA), paracetamol powder (SJZ Ruixue Pharmaceuticals, Hebei, China), citric acid monohydrate (SJZ Ruixue Pharmaceuticals, Hebei, China), amaranth (Nanjing Well Pharmaceuticals, Jiangsu, China), propylparaben sodium (Yixing Wencheng Chemical Co., Ltd., Yixing, China), methylparaben sodium (Yixing Wencheng Chemical Co., Ltd., Yixing, China), raspberry flavor (Unique Flavors and Fragrances, Kumasi, Ghana), menthol (Sigma-Aldrich, Missouri, USA), ethanol (Sigma-Aldrich), glycerol (Xi'an Henrikang Biotech Co., Ltd., Shaanxi, China), propylene glycol (Nanjing Well Pharmaceuticals, Jiangsu, China), and sorbitol powder (SJZ Ruixue Pharmaceuticals, Hebei, China). All other reagents used were of analytical grade.

### 2.2. Production of High Quality Cassava Flour (HQCF)

High quality cassava flour (HQCF) was produced as described by Dziedzoave et al. [1]. Freshly harvested cassava roots (10 to 12 months old) were peeled and washed thoroughly. The roots were then grated using a motorized grater, and the grated mash was transferred into polypropylene bags. The bags were then pressed using a manual screw press. The pressed masses were then pulverized using a motorized cassava grater into fine grits. The cassava grits were sifted, and the resulting products sun dried and milled to obtain high quality cassava flour (HQCF). The HQCF was further screened using a motorized flour sifter with a 250 *μ*m screen and then packaged into polypropylene sacks lined with transparent polythene bags.

### 2.3. Proximate Analysis and pH Determination of High Quality Cassava Flour (HQCF)

The HQCF sample was analyzed for moisture content, crude protein, crude fat, fat contents, ash, nitrogen free extract, and carbohydrate content, respectively, as described by Maziya-Dixon et al. [15].

### 2.4. Production of Glucose Syrup from HQCF Using Bacterial Alpha Amylase and Fungal Glucoamylase/Amyloglucosidase

The glucose syrup was produced from HQCF as described by Samaranayake et al. [16]. A volume of one liter of deionized water was boiled on a water bath. A mass of 400 g of HQCF was weighed into a beaker, and 400 mL deionized water was added to it while stirring to form a uniform paste. The HQCF paste was then added to the boiling water while stirring gently and continuously. A volume of 320 *μ*L bacterial alpha amylase was then added to the paste and subsequently stirred into the gelatinized paste. The temperature was maintained between 80°C and 85°C for 2 hours with constant stirring. After the 2 hours, the heat was turned down, and the liquefied starch cooled to a temperature below 60°C. Fungal glucoamylase enzyme (4 mL) was then added, and the temperature maintained between 55°C and 59°C for another 2 hours. After the 2 hours, the content was boiled for 5 to 10 minutes to deactivate the enzymes and subsequently cooled to room temperature. The resulting product was then squeezed through a cheese cloth and filtered twice using cheese cloths. Polyvinylpolypyrrolidone (12.5 mg/50 mL) was then added to the filtrate, stirred for one minute, and allowed to stand for 30 minutes. After the 30 minutes, the mixture was then centrifuged at 5000 revolutions per minute for 60 minutes and decanted. The resulting solution was then evaporated until the solution became thick and viscous.

### 2.5. Preparation of Paracetamol Syrup from HQCF

The paracetamol syrup was prepared as described by Singh et al. [17]. A mass of 2400 mg of paracetamol powder was weighed accurately into a separate beaker, and 5 mL of ethanol (96%) was added and stirred for 45 minutes. A volume of 20 mL glycerol and 10 mL propylene glycol was then added to the resulting solution. Methylparaben sodium and propylparaben sodium were weighed and added to the solution while stirring continuously. A volume of 10 mL sorbitol solution (70%) was then added and stirring continued for about 10 minutes until the solution was clear. The solution was then filtered through a sieve with a pore size of 75 *μ*m into a 100 mL volumetric flask. Sufficient quantities of amaranth red (40 mg) as well as raspberry flavor (20 *μ*L) were added as coloring and flavoring agents, respectively. The solution was then topped up to 100 mL with glucose syrup from HQCF. The final solution was transferred into a plastic bottle, capped and stored. This product was labelled as B1. The same procedure described above was used in the preparation of two other products but with the addition of sufficient quantities of sucralose as a sweetener in one product (B2) and sucrose syrup as vehicle or sweetener in the other product (B3) (Table 1).

### 2.6. Preparation of Simple Linctus

An amount of 2400 mg of citric acid monohydrate was accurately weighed into a beaker. Deionized water (10 mL) was then added and stirred with a glass rod until fully dissolved. A mass of 22 mg of menthol was weighed into a 50 mL beaker, and 5 mL of concentrated anise water was added to dissolve the menthol. The resulting solution obtained was added to the first beaker containing the dissolved citric acid monohydrate. Glycerol (20 mL) and 5 mL of propylene glycol were then added and stirred for a minute. Methylparaben sodium and propylparaben sodium were each accurately weighed and added to the solution. A volume of 20 mL of sorbitol solution (70%) was added, and the solution was stirred for 1 minute. The resulting solution was then filtered through a laboratory sieve with a pore size of 75 *μ*m into a 100 mL volumetric flask, and a sufficient quantity of amaranth (40 mg) and raspberry flavor (20 *μ*L) was added as coloring and flavoring agents, respectively. The resulting solution was then topped up to 100 mL with glucose syrup from HQCF (Table 2). The final solution was then stirred for 10 minutes, bottled and capped for storage. The same procedure described above was used in the preparation of two other products but with the addition of sufficient quantity of sucralose as a sweetener in one product (A2) and sucrose syrup as vehicle or sweetener in the other product (A3) Marriott [18].

### 2.7. Quality Assessment of Formulated Products

#### 2.7.1. Microbial Load Analysis

The formulated products as well as two brands each of paracetamol syrup and simple linctus (coded B4, B5, A4, and A5, respectively) marketed in retail pharmacies in the Kumasi metropolis were analyzed. Petri dishes were arranged according to the various samples in a laminar flow hood and labeled accordingly. The test tube rack containing the samples was also placed in the laminar flow hood, and 0.1 mL (100 *μ*L) of each of the samples was carefully pipetted into the Petri dishes after which 10 mL of the appropriate media was carefully added. The Petri dishes were then carefully swirled and allowed to stand for about 20 minutes in order for the media to solidify. This was repeated for the rest of the samples. The Petri dishes were then inverted and placed in an incubator at 37°C for 48 hours British Pharmacopoeia Commission [19].

#### 2.7.2. pH and Organoleptic Tests

The pH of the formulated products was determined using a calibrated pH meter. Organoleptic tests as well as other physical tests were carried out using the appropriate sense organs. The taste masking capacity of the glucose syrup produced from HQCF in formulations A1 and B1 were compared to that of the sucrose syrup used in the other formulations. Fifteen adults voluntarily participated in this sensory evaluation test.

#### 2.7.3. Assay of Paracetamol Syrup

The paracetamol syrup was assayed as described in the British Pharmacopoeia, 2018. An exact volume of the paracetamol syrup equivalent to 0.15 grams of paracetamol was measured into a 200 mL volumetric flask. A volume of 50 mL of 0.1 M sodium hydroxide was measured using a measuring cylinder and added to the paracetamol syrup in the 200 mL volumetric flask. The flask was then shaken for 15 minutes and topped up to the 200 mL mark with deionized water. The resulting solution was then filtered, and 10 mL was pipetted into a 100 mL volumetric flask and subsequently diluted to the mark with deionized water. An accurate volume of 10 mL of the solution was then pipetted into a 100 mL volumetric flask, and a volume of 10 mL 0.1 M sodium hydroxide was added and topped up to the 100 mL mark with deionized water. The absorbance of the resulting solution was then determined spectrophotometrically in triplicates at a wavelength of 275 nm using 0.1 M NaOH as blank. This was repeated for all the samples of the paracetamol syrup. The absorbance obtained was then inserted into the previously determined calibration equation (*y* = 724.78*x*–0.0124, *R*^2^ = 0.9971) to obtain the percentage content of paracetamol in the formulation.

#### 2.7.4. Assay of Adult Simple Linctus

The simple linctus formulations were also assayed as described in the British Pharmacopoeia, 2018. Simple linctus (10 mL) was pipetted into a conical flask, and an equal volume of distilled water was added and swirled. The solution was then titrated with 0.5 M sodium hydroxide using 3 drops of phenol red as indicator. The titration was done in triplicates for each of the five simple linctus formulations. The content of the citric acid monohydrate in each formulation was then calculated in percentage weight in volume. Prior to the main titration, the 0.5 M sodium hydroxide was standardized by titrating with 10 mL 0.5 M sulfamic acid using 3 drops of phenol red as indicator.

## 3. Results and Discussion

### 3.1. Proximate Analysis and pH Determination of High Quality Cassava Flour (HQCF)

**Figure 1:**
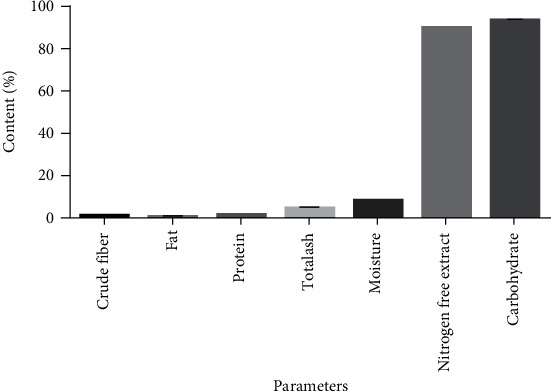
Proximate analysis of the HQCF sample (values are mean ± standard deviation, *n* = 3).

The moisture content of the extracted HQCF was found to be within the limit (˂12%) as specified by the African Organization of Standardization-ARS 840 First Edition [20] (Figure 1). Low moisture content increases the shelf life of the flour and improves the general stability as well as the microbial stability of the flour [21]. The HQCF used in this study will therefore have a prolonged shelf life and good microbial stability. The nitrogen free extract (NFE) which represents the percentage of starch and sugars present in the HQCF as indicated in Figure 1 was found to be well within the range (≥60%) specified by the African Organization of Standardization-ARS 840 First Edition [20]. HQCF are expected to have low levels of crude fiber, fat, and protein to indicate the identity and purity of the flour obtained as well as the suitability of the extraction method [22]. The crude fiber, fat, and protein contents were lesser than those reported by Iwe et al. [23] and indicate that the method used in extraction of the HQCF was appropriate. The high starch content of the HQCF used in this study facilitated its hydrolysis by the amylase enzymes in the conversion of the flour into syrup. This confirms the accession by [24] that cassava could possibly become a replacement for maize in the sweetener industry since starch from cassava can be successfully converted to various sugar syrups by hydrolysis with various enzymes. The pH values obtained for the HQCF and the respective glucose syrup were 5.52 and 6.10, respectively.

### 3.2. Quality Assessment of Formulated Products

#### 3.2.1. Microbial Quality

All formulations were free from pathogenic bacteria, namely, *Candida albicans*, *Pseudomonas aeruginosa*, *Salmonella* sp., *Escherichia coli*, and *Staphylococcus aureus* (Table 3). According to the British Pharmacopoeia Commission [19], nonsterile pharmaceutical dosage forms should be devoid of all forms of pathogenic bacteria mentioned above. Thus, all the ten formulations met this specific requirement. This indicates that the formulations used in this study were prepared under strict hygienic conditions with materials that were not contaminated. The microbial quality of the formulations can also be attributed to the high concentrations of the glucose or sucrose present in the various formulation since they tend to inhibit the growth of some microorganisms by Troja et al. [25]. Also, strict adherence to current good manufacturing practices (cGMP) played a vital role in ensuring that the formulations were devoid of any microbial contamination by Uddin et al. [26]. The preservatives used also contributed to the microbial quality of the formulations [27].

#### 3.2.2. pH and Organoleptic Tests

All the formulated products were clear solutions with their pH falling within specifications (pH 3-8) for oral solutions [28, 29]. This indicates that the syrup obtained from HQCF did not affect the final pH of the formulated product. All paracetamol syrup formulations prepared in the laboratory had red coloration with B1 (sweetened with glucose syrup from HQCF) and B3 (sweetened with sucrose syrup BP) having a sweet taste but with characteristic bitter aftertaste (Table 4). Formulation B2 (sweetened with sucralose) on the other hand had a sweet taste without any characteristic bitter aftertaste. Formulations B4 and B5 which were bought from the retail pharmacies were both magenta in color. They were sweetened with sucrose syrup as well, and as a result, they were also sweet with a characteristic bitter aftertaste. Sucralose has a relative sweetness of 600 as compared to sucrose with a relative sweetness of 1[30]. This explains why there was no characteristic bitter aftertaste in formulation B2 as compared to formulations B1, B3, B4, and B5. Ultimately, the glucose syrup produced from HQCF was able to match the taste masking property exhibited by the sucrose syrup (they all produced a sweet taste with characteristic bitter aftertaste).

The simple linctus formulations prepared in the laboratory, namely, A1, A2, and A3, were all red in color, and they all had a sweet taste (Table 4). However, formulation A2 was the sweetest amongst the three formulations because of the added sucralose which is 600 times sweeter than sucrose [30, 31]. Formulations A4 and A5 which were purchased from retail pharmacies were also sweet with A4 having a magenta color while formulation A5 was colorless. Taste masking is described as a reduction in the unpleasant taste that would have prevailed in the absence of a taste masking agent Ahire et al. [32]. The glucose syrup produced from HQCF aptly served this purpose in the paracetamol syrup and simple linctus formulations.

#### 3.2.3. Drug Content of Paracetamol Syrups and Adult Simple Linctus Formulations

**Figure 2:**
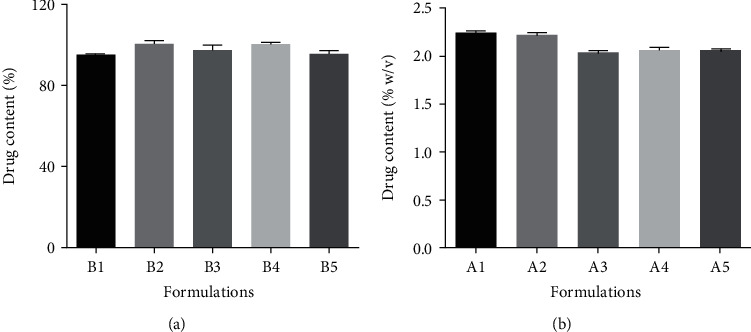
Drug content of formulations: (a) paracetamol syrups; (b) simple linctus (values are mean ± standard deviation, *n* = 3).

All the paracetamol syrup formulations passed the test for the content of active ingredient as stipulated by the British Pharmacopeia, 2018 (90% to 110% of the label claim) (Figure 2(a)). Since all formulations passed the test for the percentage drug content irrespective of the vehicle used, glucose syrup from HQCF can be used successfully in the formulation of paracetamol syrups without affecting the amount of paracetamol present in the formulation.

According to the British Pharmacopoeia Commission [19], the content of free acid calculated as citric acid monohydrate should be between 2.00% *w*/*v* to 2.65% *w*/*v*. The values obtained for all formulations were well within specifications (Figure 2(b)). These results suggest that glucose syrup produced from HQCF can be used as a vehicle in the formulation of simple linctus without altering the amount of active ingredient present in the formulation.

## 4. Conclusion

This study has revealed that glucose syrup produced from high quality cassava flour (HQCF) can be used as a vehicle in the preparation of paracetamol syrups and simple linctus formulations. Also, glucose syrup produced from HQCF can serve as a substitute for sucrose syrup in these formulations since its taste masking capacity matched that of sucrose syrup and did not affect the pharmaceutical quality of the formulated products.

## Figures and Tables

**Table 1:** Composition of formulated paracetamol syrup BP.

Ingredients	Formulation code		
	B1	B2	B3
Paracetamol	2400 mg	2400 mg	2400 mg
Propylparaben sodium	30 mg	30 mg	30 mg
Methylparaben sodium	270 mg	270 mg	270 mg
Sucralose	—	100 mg	—
Ethanol (96%)	5 mL	5 mL	5 mL
Glycerol	20 mL	20 mL	20 mL
Propylene glycol	10 mL	10 mL	10 mL
Sorbitol solution (70%)	20 mL	20 mL	20 mL
Raspberry flavor	20 *μ*L	20 *μ*L	20 *μ*L
Amaranth	40 mg	40 mg	16 mg
Glucose syrup from HQCF	100 mL	100 mL	—
Sucrose syrup	—	—	100 mL

**Table 2:** Composition of formulated simple linctus.

Ingredients	Formulation code		
	A1	A2	A3
Citric acid monohydrate	2400 mg	2400 mg	2400 mg
Menthol	22 mg	22 mg	22 mg
Methylparaben sodium	270 mg	270 mg	270 mg
Propylparaben sodium	30 mg	30 mg	30 mg
Sucralose	—	15 mg	—
Glycerol	20 mL	20 mL	20 mL
Propylene glycol	5 mL	5 mL	5 mL
Sorbitol solution (70%)	20 mL	20 mL	20 mL
Concentrated anise water	5 mL	5 mL	5 mL
Raspberry flavor	20 *μ*L	20 *μ*L	20 *μ*L
Amaranth	40 mg	40 mg	16 mg
Glucose syrup from HQCF	100 mL	100 mL	—
Sucrose syrup	—	—	100 mL

**Table 3:** Microbial load and specific media plate count of the various samples.

Sample	Media/count					
	Nutrient agar	Sabouraud agar	MacConkey agar	Mannitol agar	Cetrimide agar	Bismuth sulfite agar
A1	0	0	0	0	0	0
A2	0	0	0	0	0	0
A3	0	0	0	0	0	0
A4	0	0	0	0	0	0
A5	0	0	0	0	0	0
B1	0	0	0	0	0	0
B2	0	0	0	0	0	0
B3	0	0	0	0	0	0
B4	0	0	0	0	0	0
B5	0	0	0	0	0	0

**Table 4:** Organoleptic properties and pH of formulations.

Samples	pH	Description	Taste	Color
A1	3.82 ± 0.02	Clear solution	Sweet	Red
A2	3.84 ± 0.01	Clear solution	Very sweet	Red
A3	3.94 ± 0.01	Clear solution	Sweet	Red
A4	4.01 ± 0.02	Clear solution	Sweet	Magenta
A5	3.96 ± 0.03	Clear solution	Sweet	Colorless
B1	6.28 ± 0.02	Clear solution	Sweet +bitter aftertaste	Red
B2	6.33 ± 0.02	Clear solution	Sweet	Red
B3	7.66 ± 0.02	Clear solution	Sweet +bitter aftertaste	Red
B4	5.93 ± 0.07	Clear solution	Sweet +bitter aftertaste	Magenta
B5	5.52 ± 0.03	Clear solution	Sweet +bitter aftertaste	Magenta

## Data Availability

The data used to support the findings of this study are included in the article and also available from the corresponding author upon request.
